# Novel pneumonia score based on Systemic Immune-inflammation Index and Prognostic Nutritional Index in elderly patients

**DOI:** 10.3389/fmed.2025.1699433

**Published:** 2026-01-05

**Authors:** Xiaofei Chen, Zhijia Zhao, Yi Liang, Yujing Zhou, Huaying Wang, Wanjun Yu

**Affiliations:** Department of Respiratory and Critical Care Medicine, The Affiliated People’s Hospital of Ningbo University, Ningbo, Zhejiang, China

**Keywords:** community-acquired pneumonia, Systemic Immune-inflammation Index (SII), Prognostic Nutritional Index (PNI), mortality, elderly

## Abstract

**Background:**

The mortality rate for elderly patients with community-acquired pneumonia (CAP) admitted to intensive care units (ICU) is high. The combination assessment of Systemic Immune-inflammation Index (SII) and Prognostic Nutritional Index (PNI) can provide a more comprehensive evaluation of the patient’s immune response, systemic inflammatory burden, and nutritional metabolic status.

**Methods:**

From the Medical Information Mart for Intensive Care IV database (MIMIC-IV, version 3.1), we selected 12457 patients with CAP admitted to the ICU. After exclusions, 634 patients were included and randomly split into training (*n* = 444, 70%) and internal validation (*n* = 190, 30%). Meanwhile, an independent external validation cohort comprised 149 patients admitted to The Affiliated People’s Hospital of Ningbo University (January 2024–March 2025) was collected. Optimal thresholds for SII and PNI were derived from receiver operating characteristic (ROC) analysis in the training cohort, which were subsequently used to calculate the SII-PNI score. Model performance was evaluated through net reclassification improvement, decision-curve analysis, logistic regression analysis, and Kaplan-Meier curves. Validation was performed in internal and external cohorts to assess the model’s predictive value in geriatric CAP patients.

**Results:**

Receiver operating characteristic analysis determined the optimal cutoff values for SII (2030.28; AUC = 0.573, 95% CI 0.517–0.628, *p* < 0.05) and PNI (29.07; AUC = 0.638, 95% CI 0.584–0.692, *p* < 0.001) in the training cohort. The SII-PNI scoring model was subsequently developed using these thresholds and demonstrated predictive value for 30-day in-hospital mortality [OR: 2.19 (95% CI: 1.62–2.95), *p* < 0.001]. Kaplan-Meier survival analysis confirmed consistent prognostic performance across all cohorts, that patients with a score of 2 on the SII-PNI scale had significantly higher 30-day mortality compared to those with scores of 0 or 1 (*p* < 0.05).

**Conclusion:**

The SII-PNI may serve as a adjunct for evaluating the 30-day mortality rate among elderly ICU patients admitted with CAP.

## 1 Introduction

Age ≥ 65 years is an independent risk factor for community-acquired pneumonia (CAP) (1). Among patients with CAP aged 60 years and older, the 30-day mortality rate reaches 24%, while the 1-year mortality rate can rise to 47% (2). Additionally, approximately 16% of these patients are readmitted within 30 days following initial discharge (3, 4). This may be associated with impaired swallowing reflex, reduced mucociliary clearance, diminished immune function, and cardiopulmonary dysfunction in elderly patients (5). Therefore, for elderly patients with CAP, more effective methods for prognostic assessment are required to improve clinical management (6).

Clinical deterioration in patients with CAP predominantly occurs within the first 72 h of presentation (7), highlighting the importance of early prognostic assessment to guide timely implementation of appropriate monitoring and therapeutic interventions. The Pneumonia Severity Index (PSI) assesses the severity of pneumonia based on 20 variables encompassing demographic characteristics, clinical findings, and comorbid conditions. However, this system has certain limitations, such as operational complexity and the omission of key risk factors, including diabetes and chronic obstructive pulmonary disease (COPD). In comparison, the CURB-65 score demonstrates greater clinical utility due to its simplicity in calculation and ease of implementation. Nevertheless, it may underestimate disease severity in elderly patients with chronic comorbidities (8).

Malnutrition is an independent predictor of mortality in elderly patients with CAP. The Prognostic Nutritional Index (PNI), calculated based on serum albumin levels and lymphocyte counts, serves as a comprehensive biomarker of inflammatory response and nutritional status, demonstrating prognostic significance in both CAP (9) and gastrointestinal cancer (10). Similarly, the Systemic Immune-inflammation Index (SII), derived from platelet, neutrophil, and lymphocyte counts, serves as a quantitative measure of systemic inflammatory burden and is significantly associated with clinical outcomes in rheumatoid arthritis (11) and infectious diseases (12). Previous studies have demonstrated that the combination of SII and PNI yields synergistic effects, with the composite SII-PNI score exhibiting superior performance compared to individual indices in predicting chemotherapy response and survival outcomes among oncology patients (10, 13, 14).

While the SII and PNI have each demonstrated independent prognostic value in infection-related outcomes, their integration into a standardized risk stratification system has not been fully explored. There is currently no comprehensive evaluation of the combined SII-PNI score in predicting clinical outcomes among critically ill elderly patients with CAP. We propose that this composite biomarker could enhance the early identification of high-risk individuals through the simultaneous assessment of systemic inflammatory response and nutritional status.

## 2 Materials and methods

### 2.1 Data source and ethics statement

This retrospective study utilized data from the Medical Information Mart for Intensive Care IV (MIMIC-IV, version 3.1) database^1^, a publicly accessible repository containing de-identified clinical data of patients admitted to the emergency department or intensive care units (ICU) at Beth Israel Deaconess Medical Center in Boston, Massachusetts, USA. This comprehensive database contains granular clinical data, including demographic characteristics, vital signs, medication administration records, laboratory test results, ICD-coded diagnoses, therapeutic interventions, and survival outcomes. Xiaofei Chen (ID: 14360095) has access to the database and was responsible for the data extraction.

For the external validation cohort, patients (age > 65) with CAP admitted to the ICU at The Affiliated People’s Hospital of Ningbo University were retrospectively included between January 2024 and March 2025. This study protocol received ethical approval from the Ethics Committee of the Affiliated People’s Hospital of Ningbo University [2024-006] following the Declaration of Helsinki principles.

### 2.2 Study population

**FIGURE 1:**
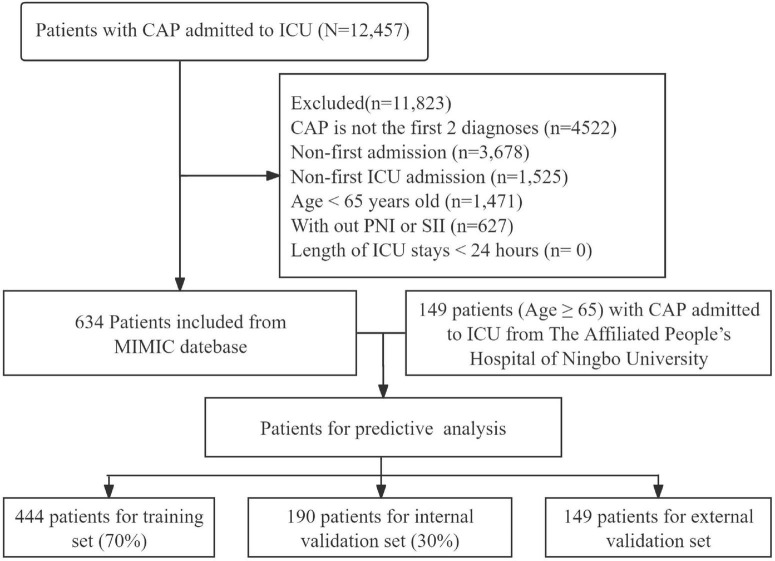
Flowchart of the study. CAP, community-acquired pneumonia; ICU, intensive care unit; SIIS, Systemic Immune-inflammation Index; PNI, Prognostic Nutritional Index.

In this study, we selected 12457 patients with CAP admitted to the ICU from MIMIC-IV database. After exclusions, 634 patients were included and randomly split into training (*n* = 444, 70%) and internal validation (*n* = 190, 30%) (Figure 1). Laboratory data for patients with CAP within the first 24 h of ICU admission were extracted. Patients were excluded based on the following criteria: (1) CAP is not the first 2 diagnoses; (2) patients non-first admission, for whom only the initial stay was included; (3) patients non-first ICU admission; (4) patients aged less than 65 years old; (5) the absence of a blood routine test makes it impossible to calculate SII and PNI; (6) patients stayed in ICU shorter than 24 h (Figure 1). A total of 149 patients with CAP admitted to the ICU at The Affiliated People’s Hospital of Ningbo University were enrolled as an external validation cohort.

### 2.3 Statistical analysis

Two prognostic indices were calculated: PNI = 10 × serum albumin (g/dL) + 5 × lymphocyte count (10^9^/L); SII = platelet count (10^9^/L) × (neutrophil count/lymphocyte count) (13).

Data normality was assessed using Shapiro-Wilk tests. Continuous variables with normal distribution were presented as mean ± SD, while non-normally distributed variables were reported as median [interquartile range (IQR)]. Between-group comparisons employed: (1) χ^2^ tests for categorical variables; (2) Student’s *t*-tests for normally distributed continuous variables; and (3) Wilcoxon rank-sum test for non-parametric distributions.

The prognostic performance of SII and PNI for 30-day in-hospital mortality was evaluated using receiver operating characteristic (ROC) analysis. Optimal thresholds were established using Youden’s index, from which sensitivity, specificity, and area under the curve (AUC) were derived. These thresholds were subsequently used to calculate the SII-PNI score, as reported in previous studies (15). The prediction performance of SII-PNI and existing scoring models was compared using net reclassification improvement, decision-curve analysis. Univariate and multivariable logistic regression assessed associations between the SII-PNI score and adverse hospitalization outcomes. Multivariable models adjusted for age, gender, temperature, systolic and diastolic blood pressure, prothrombin time, blood urea nitrogen, creatinine, glucose, and total bilirubin. Survival distributions were evaluated with Kaplan-Meier analysis.

Statistical analyses were performed using SPSS 25.0 (IBM Corporation, United States) and R software 4.3.2 (R Foundation for Statistical Computing, Austria). Statistical significance was determined using a two-sided *p*-value threshold of 0.05.

## 3 Results

### 3.1 Demographic characteristics

The training cohort included 444 patients, comprising 278 (62.6%) survivors and 166 (37.4%) non-survivors (Table 1). Analysis demonstrated that the SII was higher in the non-survival group compared to survivors (2591.0 vs. 1878.18, *p* < 0.05), while the PNI was lower (31.38 vs. 34.95, *p* < 0.001). Although the study population consisted of elderly individuals (>65 years), the non-survival group was significantly older than the survival group (78 vs. 70, *p* < 0.001). Furthermore, patients in the non-survival group exhibited reduced blood lymphocyte counts (0.77 vs. 0.94, *p* < 0.05) and albumin levels (2.7 vs. 3.0, *p* < 0.001) relative to survivors.

**TABLE 1:** Baseline characteristics of the training cohort between survivors and non-survivors.

Variables	Survivors (*n* = 278)	Non-survivors (*n* = 166)	Total (*n* = 444)	*p*
Age, year	70 (68, 80)	78 (71, 84)	75 (69, 81)	<0.001
Gender (*n*, %)				0.557
Female	121 (43.53%)	77 (46.39%)	198 (44.59%)	
Male	157 (56.47%)	89 (53.61%)	246 (55.41%)	
Respiratory rate, bpm	22 (18, 26)	21 (18, 27)	22 (18, 27)	0.742
Temperature	36.86 (36.44, 37.28)	36.69 (36.21, 37.10)	36.78 (36.39, 37.17)	<0.001
SBP, mmHg	126 (107, 145)	116 (100, 134)	122 (104, 141)	0.001
DBP, mmHg	68 (58, 84)	62 (53, 76)	66 (56, 80)	<0.001
Heart rate, bpm	90 (77, 106)	93 (79, 104)	92 (78, 105)	0.219
**Comorbidities (*n*, %)**				
Chronic pulmonary disease	130 (46.76%)	63 (37.95%)	193 (43.37%)	0.07
Congestive heart failure	133 (47.84%)	74 (44.58%)	207 (46.62%)	0.505
Diabetes	107 (38.49%)	50 (30.12%)	157 (35.36%)	0.074
Hypertension	97 (34.89%)	53 (31.93%)	150 (33.78%)	0.523
Renal diseases	95 (34.17%)	53 (31.93%)	148 (33.33%)	0.627
**Laboratory results**				
Lymphocytes (× 10^9^/L)	0.94 (0.51, 1.43)	0.77 (0.48, 1.27)	0.87 (0.50, 1.36)	<0.05
Neutrophils (× 10^9^/L)	9.23 (5.94, 14.38)	10.07 (7.13, 15.71)	9.54 (6.31, 14.53)	0.093
Platelet (× 10^9^/L)	196.00 (141.75, 262.50)	199.00 (129.75, 291.00)	197.00 (138.00, 267.00)	0.920
Albumin (g/dL)	3.00 (2.60, 3.30)	2.70 (2.28, 3.10)	2.90 (2.50, 3.20)	<0.001
Creatinine (mg/dL)	1.10 (0.90, 2.00)	1.20 (0.90, 2.43)	1.2 (0.90, 2.10)	0.188
BUN (mg/dL)	29.00 (18.00, 47.00)	33.00 (20.75, 57.25)	30.00 (19.00, 50.75)	0.091
Glucose (mg/dL)	130.50 (107.75, 179.00)	128.50 (106.00, 174.00)	130.00 (107.00, 177.00)	0.485
Total bilirubin (mg/dL)	0.60 (0.40, 0.93)	0.50 (0.30, 1.20)	0.60 (0.40, 1.00)	0.578
PT (s)	14.10 (12.50, 16.70)	14.80 (13.08, 18.60)	14.30 (12.70, 17.30)	<0.05
SII	1878.18 (937.97, 3986.87)	2591.09 (1250.44, 5177.80)	2182.83 (1059.83, 4507.92)	<0.05
PNI	34.95 (30.38, 39.03)	31.38 (26.68, 36.34)	33.73 (28.90, 38.24)	<0.001
**Scoring systems**				
SAPS-II	43 (35, 51)	49 (39, 59)	45 (36, 55)	<0.001
SOFA	7 (5, 10)	9 (6, 12)	8 (5, 11)	<0.001
SIRS	3 (2, 3)	3 (2, 3)	3 (2, 3)	<0.05
**Therapy (*n*, %)**				
Glucocorticoids	67 (24.10%)	56 (33.73%)	123 (27.70%)	<0.05
CRRT	28 (10.07%)	30 (18.07%)	58 (13.06%)	<0.05
Invasive ventilation	184 (66.19%)	110 (66.27%)	294(66.22%)	0.987

Data following a normal distribution are shows the mean ± SD, while those not normally distributed are depicted as median (IQR) and categorical variables are displayed as count (%). SBP, systolic blood pressure; DBP, diastole blood pressure; PT, prothrombin time; SAPS-II, simplified acute physiology score; SOFA, sequential organ failure assessment score; SIRS, systemic inflammatory response syndrome score; CRRT, Continuous renal replacement therapy.

### 3.2 Associations between PNI or SII and prognosis

**FIGURE 2:**
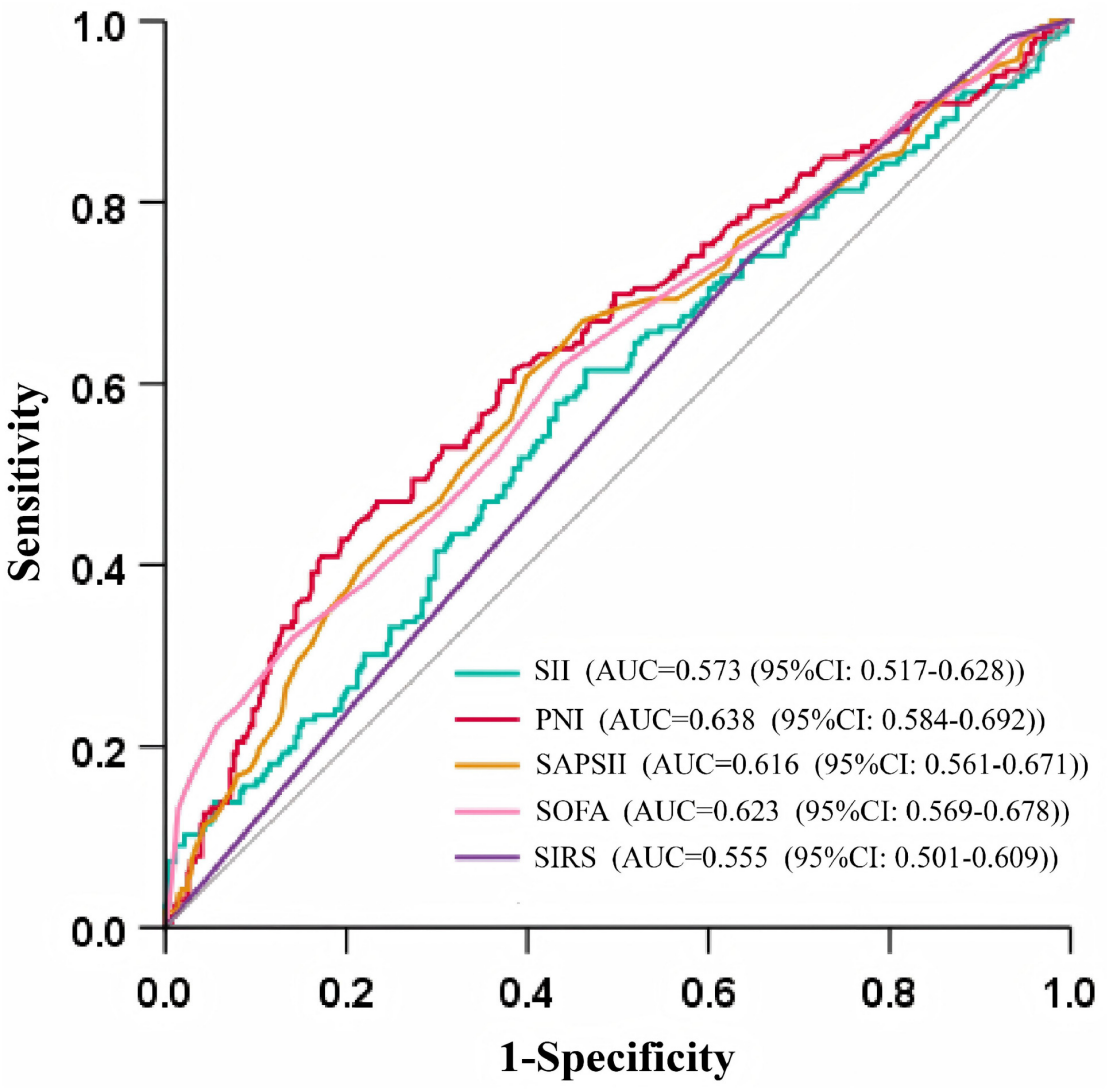
ROC curves for predicting 30-day in-hospital mortality.

Figure 2 presents ROC curves comparing the prognostic performance of SII, PNI, SAPS-II, SOFA, and SIRS in predicting 30-day in-hospital mortality. PNI demonstrated superior diagnostic accuracy, with an AUC of 0.638 (95% CI: 0.584–0.692; *p* < 0.001), compared to an AUC of 0.573 (95% CI: 0.517–0.628; *p* < 0.05) for SII. Optimal cutoff values determined by Youden’s index were 2030.28 for SII (sensitivity 61.4%, specificity 53.6%) and 29.07 for PNI (sensitivity 41.0%, specificity 82.7%).

### 3.3 Survival outcomes with different SII-PNI scores

According to previous studies (13), we defined the SII-PNI score using optimal cutoffs (SII: 2030.28; PNI: 29.07) to stratify patients: 1 point was assigned if SII ≥ 2030.28, and 0 points if SII < 2030.28; 1 point was assigned if PNI ≤ 29.07, and 0 points if PNI > 29.07. The SII-PNI score (ranging from 0 to 2) was the sum of these two individual scores. Baseline characteristics stratified by SII-PNI are presented in Table 2. After stratifying the training cohort according to SII-PNI scores, no significant differences were observed in age or gender distribution across the three groups (*p* > 0.05). However, patients with an SII-PNI score of 2 had significantly higher SAPS-II, SOFA, and SIRS scores compared to those with scores of 0 or 1 (*p* < 0.05). Additionally, this group demonstrated a significantly higher 30-day in-hospital mortality rate than the score 0 and score 1 groups (*p* < 0.05), as well as longer ICU length of stay compared to patients with a score of 0 (*p* < 0.05).

**TABLE 2:** Baseline characteristics of the study cohort stratified by SII-PNI group.

Variables	SII-PNI = 0 (*n* = 176)	SII-PNI = 1 (*n* = 189)	SII-PNI = 2 (*n* = 79)	*p*
Age, year	75 (68, 82)	75 (69, 81)	76 (71, 82)	0.599
Gender (*n*, %)				0.749
Female	75 (42.6%)	88 (46.5%)	35 (44.3%)	
Male	101 (57.3%)	101 (53.4%)	44 (55.6%)	
**Comorbidities (*n*, %)**				
Chronic pulmonary disease	63 (35.7%)	94 (49.7%)	35 (44.3%)	0.027
Congestive heart failure	82 (46.5%)	95 (50.2%)	30 (37.9%)	0.184
Diabetes	58 (32.9%)	74 (39.1%)	25 (31.6%)	0.348
Hypertension	58 (33.5%)	65 (34.3%)	26 (32.9%)	0.950
Renal diseases	62 (35.2%)	65 (34.3%)	21 (26.5%)	0.368
SII	937.55 (514.41, 1441.48)	3371.94 (2220.22, 5810.70)	5352.67 (3277.24, 10308.72)	<0.001
PNI	37.14 (33.83, 40.69)	33.35 (29.64, 36.59)	25.50 (23.03, 27.49)	<0.001
**Scoring systems**				
SAPS-II	45 (35, 54)	43 (36, 54)	49 (38, 59)*#	0.056
SOFA	7 (6, 11)	7 (4, 11)	9 (6, 12)*#	<0.05
SIRS	3 (2, 3)	3 (2, 3)	3 (3, 4)*#	<0.05
**Clinical outcomes**				
LOS ICU (day)	4.78 (2.22, 10.01)	5.91 (2.65, 11.21)	6.44 (2.97, 13.57)*	0.077
LOS hospital (day)	13.94 (7.83, 21.52)	13.67 (7.21, 22.45)	14.90 (7.74, 25.51)	0.727
30-day mortality (*n*, %)	45 (25.56%)	72 (38.10%)	49 (62.02%)*#	<0.001

SAPS-II, simplified acute physiology score; SOFA, sequential organ failure assessment score; SIRS, systemic inflammatory response syndrome score; LOS, length of stay; ICU, intensive care unit.

*: vs. SII-PNI = 0, *P* < 0.05;

#: vs. SII-PNI = 1, *P* < 0.05.

### 3.4 Model performance

**FIGURE 3:**
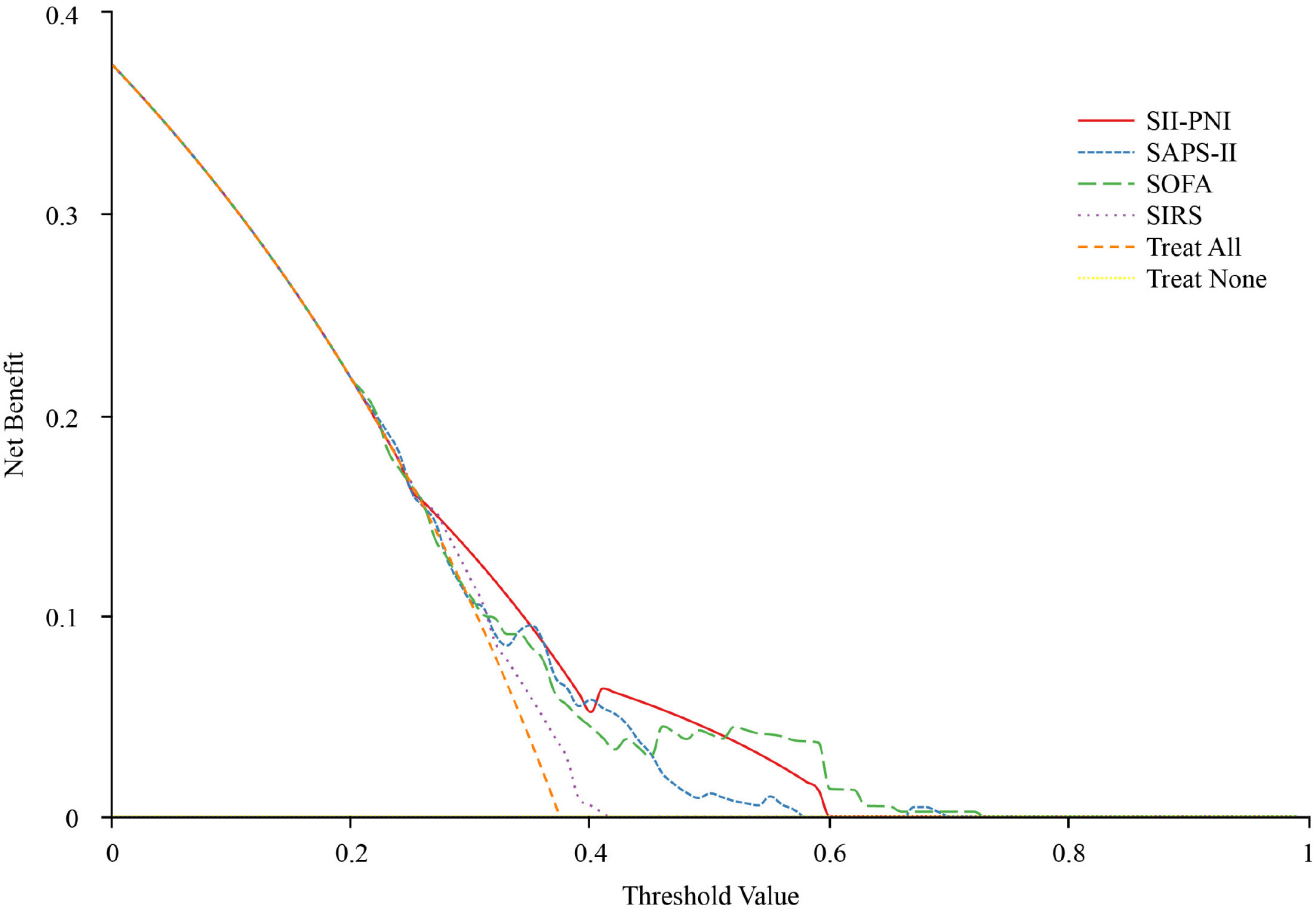
Decision-curve analysis of SII-PNI, SOFA, SAPS-II and SIRS model.

In the net reclassification improvement analysis, the predictive performance of the SII-PNI model was comparable to that of the SOFA model (NRI = 0.137, *p* = 0.161), while it demonstrated superior discriminatory ability relative to the SAPS-II (NRI = 0.308, *p* = 0.0.001) and SIRS (NRI = 0.349, *p* < 0.0.001) models. Decision curve analysis further indicated that the SII-PNI model provides clinically meaningful prognostic information for evaluating outcomes in patients with community-acquired pneumonia (Figure 3).

### 3.5 Logistic regression analysis and subgroup analysis

Possible confounding factors were identified through a univariate logistic regression and then verified in a multivariate logistic regression to evaluate the influence of these confounding factors on the model. Multivariable logistic regression analyses (Table 3) demonstrated that the SII-PNI score was an independent predictor of 30-day mortality across progressively adjusted models. In the unadjusted model, each one-unit increase in the SII-PNI score was associated with a 2.13-fold higher risk of mortality (95% CI 1.61–2.81; *p* < 0.001). This association persisted after adjustment for demographic characteristics, hemodynamic parameters, and biochemical markers.

**TABLE 3:** Multivariable logistic regression for 30-day in-hospital mortality.

Models	OR	95% CI	*p*
Model 1	2.13	1.61–2.81	<0.001
Model 2	2.09	1.56–2.80	<0.001
Model 3	2.19	1.62–2.95	<0.001

Model 1: unadjusted. Model 2: adjusted for gender, age, temperature, SBP, DBP. Model 3: adjusted for gender, age, temperature, SBP, DBP, PT, BUN, creatinine, glucose, total bilirubin. SBP, systolic blood pressure; DBP, diastole blood pressure; PT, prothrombin time; BUN, blood urea nitrogen.

**FIGURE 4:**
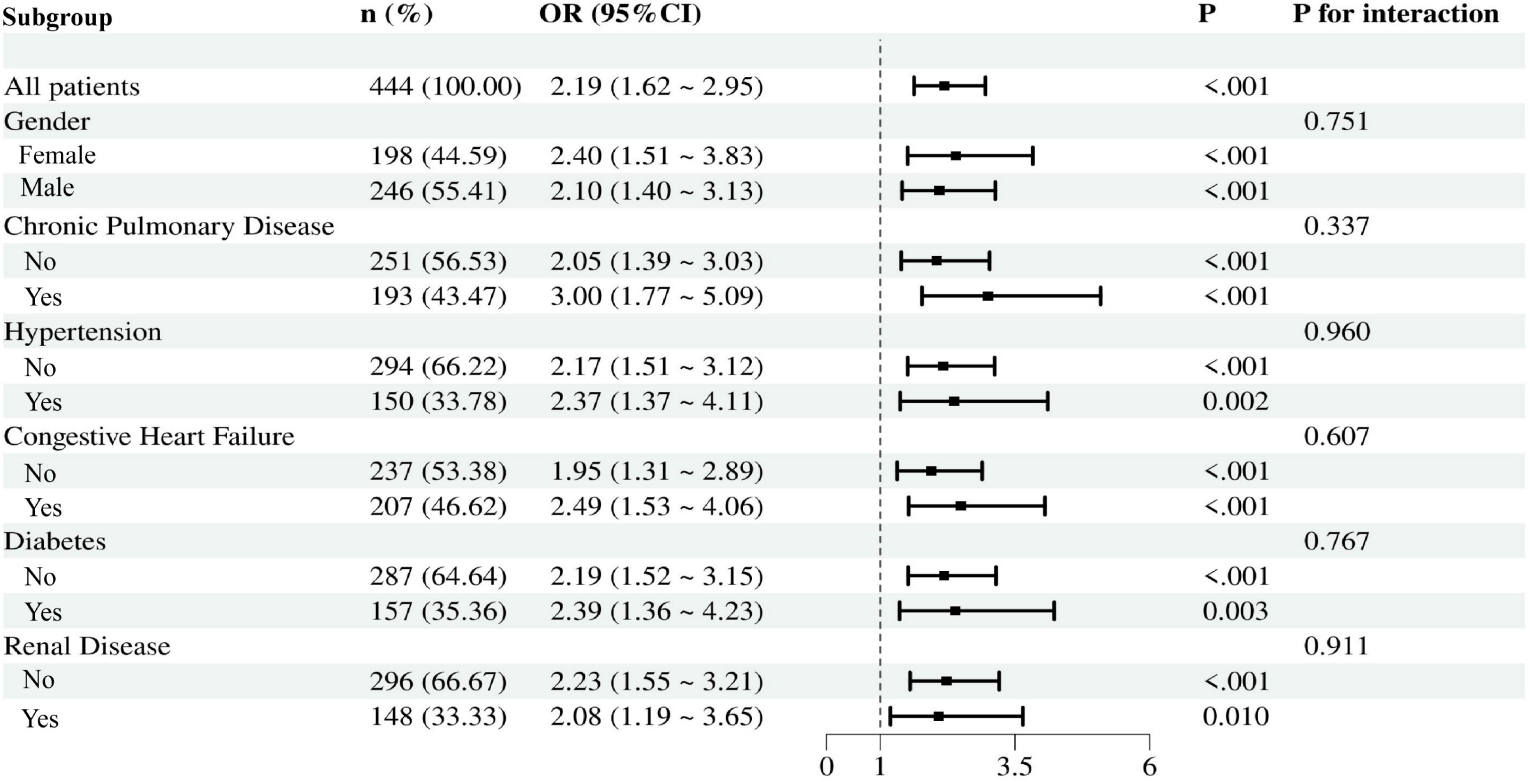
Forest plot of ORs for the 30-day in-hospital mortality in different subgroups. OR is adjusted for age, temperature, SBP, DBP, PT, BUN, creatinine, glucose and total bilirubin. SBP, systolic blood pressure; DBP, diastole blood pressure; PT, prothrombin time; BUN, blood urea nitrogen.

The prognostic value of SII-PNI for 30-day in-hospital mortality was further evaluated across various patient subgroups, including those defined by gender and comorbidities such as renal disease, diabetes, chronic pulmonary disease, hypertension, and congestive heart failure (Figure 4). The analysis revealed that higher SII-PNI scores were consistently associated with increased mortality risk across all subgroups.

### 3.6 Model validation

**FIGURE 5:**
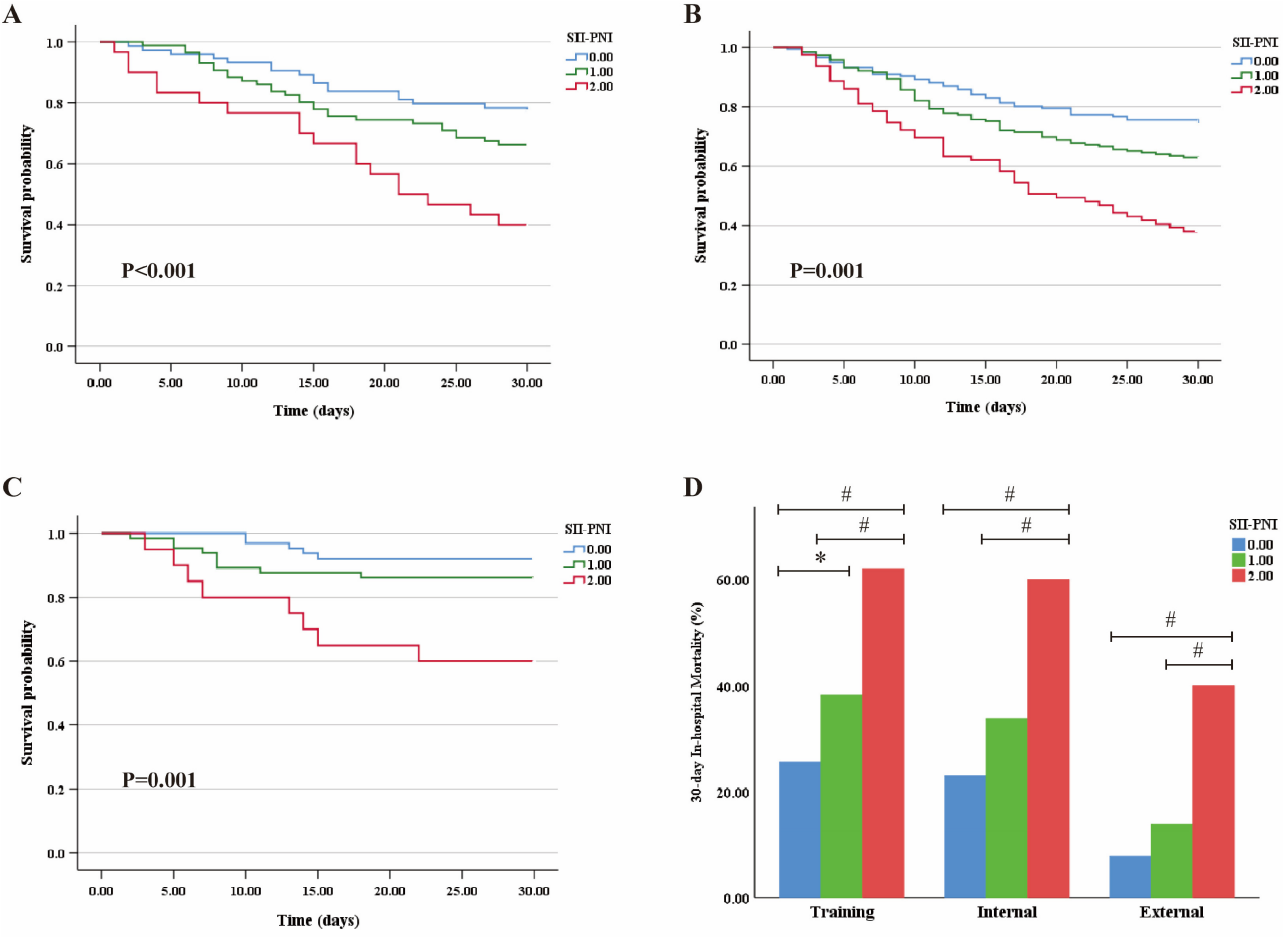
Kaplan-Meier survival curves for 30-day in-hospital mortality for patients with different SII-PNI score in different cohorts. **(A)** Training cohort; **(B)** internal cohort; **(C)** external cohort; **(D)** illustrates the 30-day in-hospital mortality rates among patients stratified by SII-PNI scores in the training, internal validation, and external validation cohorts; *: vs. SII-PNI = 1, *P* < 0.05; #: vs. SII-PNI = 2, *P* < 0.05.

To evaluate the prognostic value of the SII-PNI score for 30-day in-hospital mortality among elderly patients with CAP admitted to the ICU, Kaplan-Meier survival analyses were conducted in the training, internal, and external cohorts (Figures 5A–C). A higher SII-PNI score was significantly associated with poorer clinical outcomes (*p* < 0.05). Patients with an SII-PNI score of 2 consistently exhibited higher mortality risk compared to those with scores of 0 or 1 (Figure 5D).

## 4 Discussion

Hospitalized patients with CAP demonstrate significant age-dependent disparity in mortality, with rates of 2.8% observed in in individuals under 60 years of age compared to 26.8% in those aged 60 years and older (16). This substantial difference in mortality underscores the critical need for a clinically practical prognostic tool to facilitate effective risk stratification of elderly CAP patients in ICU. In response, we developed and validated a novel prediction model for 30-day in-hospital mortality using data from the MIMIC-IV database and electronic medical records of a tertiary hospital in China. The model incorporates four routinely measured biomarkers (neutrophil count, lymphocyte count, platelet count, and serum albumin) readily available within the first 24 h of ICU admission. Validation analyses demonstrated robust predictive performance in both internal and external cohorts.

The dynamic balance between pro-inflammatory and anti-inflammatory systems plays a critical role in determining the clinical outcomes of patients. Routine blood tests provide a variety of hematological parameters, such as neutrophils, lymphocytes, and platelets, which can reflect immune responses and inflammatory states. Previous studies have demonstrated that lymphopenia is associated with elevated short- and long-term mortality in patients with CAP (17). This association may be explained by infection-induced immune dysregulation, characterized by aberrant activation of specific immune cell populations, dysregulated cytokine secretion, and subsequent activation of the apoptotic pathway, all of which contribute to impaired inflammatory responses (18). These immunological alterations can lead to an immunosuppressive state, thereby worsening disease severity and increasing the risk of mortality. Neutrophils are the primary effector cells that participate in innate immunity during microbial invasion. While essential for pathogen elimination, excessive or uncontrolled neutrophil activation results in the excessive release of cytotoxic components, including proteolytic enzymes, reactive oxygen species, cationic polypeptides, and proinflammatory cytokines. This cascade of inflammatory mediators induces tissue damage (19). A hyperinflammatory state may also disrupt normal coagulation processes, promote immune-mediated thrombosis, and lead to disseminated intravascular coagulation, ultimately compromising microcirculatory homeostasis (20). Notably, neutrophil function declines with age, accompanied by reduced migratory accuracy (21, 22). In older adults, pre-existing neutrophil dysfunction can be further aggravated by pulmonary infections (23). Dysregulated neutrophil activity has been significantly correlated with increased mortality and the development of severe complications, including sepsis and acute respiratory distress syndrome (24, 25). During acute infection, platelets become activated and aggregate, contributing to the coordination of inflammatory responses through interactions with monocytes, neutrophils, and vascular endothelial cells (26), while also releasing various procoagulant and pro-inflammatory mediators. While this platelet-driven amplification mechanism supports host defense, its overactivation can become pathological, leading to microcirculatory impairment and multiorgan dysfunction (27). Clinically, thrombocytosis upon hospital admission has been identified as an independent predictor of 30-day mortality in CAP, suggesting that platelet monitoring may improve risk stratification (28).

However, the prognostic evaluation of pneumonia patients based on a single routine blood parameter exhibits limited clinical reliability. The combined assessment of platelets, neutrophils, and lymphocytes provides a more comprehensive reflection of patients’ immune status and systemic inflammatory response (29). Previous studies have investigated the prognostic value of the platelet-to-lymphocyte ratio (PLR) and neutrophil-to-lymphocyte ratio (NLR) in CAP. Nevertheless, these indices have shown limited predictive accuracy in predicting in-hospital mortality (30, 31). In contrast, the SII, which integrates both NLR and PLR, has been suggested as a more robust predictor of 28-day mortality in patients with CAP (32). However, according to our findings, the SII demonstrated limited utility in predicting 30-day mortality among elderly patients with severe CAP. At the optimal cutoff value identified (SII = 2030.28), the model exhibited modest discriminative ability (AUC: 0.573, 95% CI: 0.517–0.628; *p* < 0.05), along with suboptimal classification performance (sensitivity 61.4%, specificity 53.6%). These results indicate that the SII lacks sufficient predictive accuracy for effective mortality risk stratification in this high-risk population.

In elderly patients with CAP, although systemic inflammation and immune responses necessitate close monitoring, the assessment of nutritional status is equally critical for comprehensive patient management. We observed that the PNI demonstrated significantly higher specificity (82.7% vs. 53.6%) compared to the SII in predicting mortality, albeit with lower sensitivity (41.0% vs. 61.4%). PNI is calculated based on platelet count and albumin levels, with albumin playing a pivotal role in maintaining oncotic pressure, antioxidation, and anti-inflammation (33). Clinical evidence indicates an inverse association between hypoalbuminemia and survival outcomes in critically ill patients (34). And albumin replacement therapy has been shown to confer a significant survival benefit in septic shock (35). Previous studies have demonstrated that PNI is inversely associated with all-cause mortality in CAP (36) and exhibits superior prognostic accuracy compared to the CURB-65 scoring system (9). Therefore, PNI serves as a valuable composite biomarker that simultaneously reflects both systemic inflammatory activity and nutritional status.

The integration of the SII and PNI offers a comprehensive assessment of patients’ immune function, systemic inflammatory status, and nutritional-metabolic condition. Existing research has predominantly applied this composite index to predict outcomes in oncology populations, with evidence indicating that elevated SII levels and reduced PNI values are significantly associated with adverse clinical prognosis in cancer patients (13, 15, 37). Building on established methodologies (15), we adopted the SII-PNI scoring system to evaluate outcomes in elderly patients with CAP admitted to ICU. Our results demonstrated that, in the training cohort, the SII-PNI score effectively predicted 30-day in-hospital mortality. Patients with a score of 2 exhibited significantly higher mortality rates compared to those with scores of 0 or 1 (*p* < 0.05). We further compared the predictive performance of the SII-PNI, SOFA, SAPS-II, and SIRS models. The results indicate that the SII-PNI model exhibits comparable predictive accuracy to the SOFA model (NRI = 0.137, *p* = 0.161), while outperforming both the SAPS-II (NRI = 0.308, *p* = 0.001) and SIRS (NRI = 0.349, *p* < 0.001) models. These findings are further supported by decision curve analysis (Figure 3). However, the SOFA model involves a more complex calculation process and requires a greater number of clinical parameters, limiting its practicality in routine clinical settings. In contrast, the SII-PNI model relies only on readily available laboratory markers—neutrophil count, lymphocyte count, platelet count, and albumin level—and can be easily computed, thereby offering a more convenient and clinically feasible alternative. To validate the robustness of the SII-PNI model in prognostic stratification, internal and external validation cohorts were established. Consistent findings across both validation sets confirm the reliability and predictive accuracy of the SII-PNI scoring system in assessing clinical outcomes among this high-risk patient population.

There are several limitations to this research. First, as a retrospective analysis, despite rigorous multivariable adjustments and subgroup analyses, the potential influence of unmeasured confounding factors cannot be entirely ruled out. To enhance the robustness of the scoring model, both internal and external validation strategies were implemented. Second, the study cohort included 783 patients aggregated from two distinct sources: the MIMIC-IV database and a tertiary hospital in China. While this dual-source design contributes to data diversity, the moderate sample size (*n* = 783) may limit the generalizability of the findings, particularly considering potential variations in ICU admission criteria and treatment protocols across different healthcare systems. Finally, the exclusion of cases with incomplete clinical records may have introduced selection bias. While the SII-PNI index demonstrated prognostic value in this retrospective analysis, its clinical applicability warrants confirmation through prospective, multicenter cohort studies.

## 5 Conclusion

The SII-PNI score may serve as a simple and effective predictive model for early assessment of 30-day mortality risk in elderly patients with CAP requiring ICU admission. This composite biomarker incorporates both inflammatory and nutritional parameters, thereby providing a more comprehensive pathophysiological evaluation, which may facilitate the development of personalized risk management strategies.

## Data Availability

Publicly available datasets were analyzed in this study. This data can be found here: https://physionet.org/content/mimiciv/3.1/.

## Footnotes

1
https://physionet.org/content/mimiciv/3.1/
